# Public Preference for Pet-Rabies Prophylaxis: Opportunities and Information Dissemination

**DOI:** 10.3390/tropicalmed2030046

**Published:** 2017-09-13

**Authors:** Maria B. Palamar, Maria T. Correa, Nils M. Peterson, Christopher S. DePerno

**Affiliations:** 1 NC Wildlife Resources Commission, 1751 Varsity Dr., Raleigh, NC 27606, USA; 2 NC State University College of Veterinary Medicine, 1060 William Moore Dr., Raleigh, NC 27606, USA; correa@ncsu.edu; 3 NC State University College of Natural Resources, Fisheries and Wildlife Conservation Biology, Raleigh, NC 27606, USA; nils_peterson@ncsu.edu (N.M.P.); chris_deperno@ncsu.edu (C.S.D.)

**Keywords:** rabies, rabies prevention, public health outreach, race, ethnicity

## Abstract

Risky human behavior and high density of rabies vectors in urban environments combine to increase the risk of rabies. Pet vaccination, wildlife vector management, and public health education may be the most efficient ways to prevent urban rabies epidemics. Racial, ethnic, and socio-economic factors influence the use of low-cost rabies vaccination clinics, understanding rabies reporting requirements, and learning preferences. In collaboration with the City of Greensboro and Animal Control in Guilford County, NC, we conducted a survey of rabies prevention and transmission across socio-economic strata representing Latinos, African Americans, and Whites, and different income and education levels. Compliance with vaccination was low among Latinos; African Americans and Latinos were not aware of low-cost rabies vaccination clinics; and most respondents were willing to report rabid animals but did not know whom to call. White respondents preferred online information delivery, whereas Latinos and African Americans preferred postal mail. Communication targeting the public requires the consideration of different message decoding and interpretation based on the ethnicity, income, and educational level, and other barriers such as language. Differing message delivery methods may be required to achieve full dissemination.

## 1. Introduction

Several factors are combined to make urban environments ideal for the spread of zoonotic diseases [1]. First, human population densities are highest in urban areas. Second, population densities of wildlife species which serve as rabies vectors (e.g., raccoons, foxes, and coyotes) are often highest in urban areas because these species are highly adaptable and take advantage of anthropogenic factors (e.g., garbage, denning sites in buildings, green areas protected of larger predators) that facilitate high reproduction rates and increased survival [2]. Third, pet densities are highest in urban areas. Finally, human behaviors in urban areas, such as attempting to help approachable animals, feeding wildlife purposively or not (e.g., feeding corn to raccoons, leaving pets’ food outside), increases the risk of disease transmission in urban environments. This may happen by directly relating to wildlife or by attracting a larger number of animals to a particular area.

Rabies is an important zoonosis that affects wild and domestic animals, as well as humans. The current rabies epidemic in the eastern United States is associated with a raccoon (*Procyon lotor*) variant of the virus [3]. Raccoons are widespread throughout North America and are present in high densities in urban environments [4,5]. They are well adapted to urban and suburban areas, using human housing to den, and garbage, pet food, plants in residential gardens, and urban proximate crops as a food sources [6]. These animals are hosts to a large number of pathogens (e.g., *Leptospira interrogans*, and canine distemper, rabies, and feline panleukopenia viruses) that can infect other animal species and humans [7]. Therefore, interactions between raccoons, humans, and their companion animals have led to increasing public health concerns [6]. 

Pet vaccination, wildlife vector management, and public health education may be the most efficient ways to prevent a rabies epidemic in urban environments [8]. Public health officials in Greensboro, NC, indicated that compliance with rabies vaccination laws is low. For example, of the 11 cases of pets that had been in contact with a suspected rabid animal in 2012, 10 had to be euthanized or quarantined because they did not have the proper rabies vaccination [9]. Although rabies vaccination by professionals is required by law for dogs, cats, and ferrets, and is reportable in NC, rabies immunization and reporting is difficult to enforce and the number of unvaccinated animals is difficult to estimate. There has not been any other comparable reporting method in place.

Cultural norms may contribute to challenges with pet immunization efforts. Ethnic minorities—particularly Hispanic/Latinos—are increasing in the United States, and are becoming a critically important focus of wildlife management and outreach programs [10]. Engaging minorities in wildlife management and public health programs requires the creation of language-appropriate, culturally-sensitive, and relevant information to be delivered in an accepted format and through media outlets favored by the target population. Compared to Whites, Hispanic/Latinos and African Americans know less about the signs of rabies infection in animals, transmission routes, wildlife vectors, and about first response after rabies exposure [11]. Health and illness perception, risk-taking or -aversion behaviors, and lack of access to information, may increase exposure to rabies or the reporting of suspected rabid animals [11]. The challenge of reaching minorities has been documented in other public health areas, including sexually transmitted diseases (STDs) and oral health [12]. 

Guilford County, NC, USA was a good place to find solutions for some of these challenges. In 2006, Guilford County had 37 confirmed cases of wild animal rabies, and 20 of these cases were raccoons (others included one cat, one coyote, two bats, four foxes, and five skunks). In addition, Guilford County animal control officials were anecdotally reporting a low turnout of ethnic minorities to their pet rabies vaccination clinics. After the 2006 rabies report, Guilford County Environmental Health officers were concerned with raccoon population dynamics in urban areas and in particular in Greensboro (a major city in the county), and decided to reach out to North Carolina State University to express the need for public health research. Their concern also extended to public awareness, understanding of rabies transmission, symptoms, and early treatment, as well as low-cost options for pet vaccination. The city has a total population of 237,423, of whom Hispanic/Latinos (15,412) and African Americans (88,587) account for roughly half of the population [13]. This diverse population made the city a good case study to answer some of the concerns presented by Environmental Health officials and to gain insight about the public’s knowledge, get an estimate of pet vaccination status, and understand how people would respond to an encounter with a rabid animal. Therefore, we conducted a bilingual (English/Spanish) survey in Greensboro, NC, to determine pet vaccination compliance and awareness and use of low-cost rabies vaccination clinics. Information about the understanding of the requirements for reporting suspected rabid animals (e.g., who they would report to) and differing public preferences for communication content, format, and delivery of rabies information were elicited from survey participants.

## 2. Materials and Methods

Four neighborhoods located within the northwest quadrant of the city of Greensboro were selected for the survey, given the high number of rabies-positive raccoon cases reported in 2006 and 2007 [14]. We conducted the survey during October and November 2009. We interviewed the adult (18 years or older) who answered the door in every third dwelling in each of the selected neighborhoods. If nobody answered or the person who answered refused to participate in the survey in the selected house, the next dwelling was considered the replacement. This systematic sampling strategy ensured we engaged diverse audiences often left out of sample frames based on phone numbers or formal mailing addresses [15,16]. Surveys were conducted on weekdays and weekends, alternating mornings and afternoons to decrease possible bias associated with sampling weekday and time. A total of nine interviewers participated, four males and five females, who worked in pairs with at least one bilingual interviewer in each pair. All interviewers were trained by the primary author, and carried English and Spanish copies of the questionnaire. Each respondent was asked which language (English or Spanish) they preferred; when Spanish was chosen, the respondent was asked if they wanted to talk to one of the bilingual interviewers.

**Survey design and administration**—An English version of the survey was designed and translated into Spanish by two of the authors who are bilingual. The survey was then back-translated to English in order to check for semantic and conceptual equivalence. Information was elicited on: learning about rabies, attitudes towards and reporting on rabies, pet-vaccination status, demographic and socio-economic status i.e., previous year’s income classified into categories of 0 (≤$14,999) to 8 (≥$60,000); age; education, categorised as 0 (grammar school) to 4 (graduate degree); years of residence in the area; number of household residents; and gender and ethnicity. Ethnicity included Hispanic/Latino and race options were White, Asian, Black or African American, Native American, and Hawaiian or other Pacific Islander as defined in US Census Bureau [17]. Twelve questions (12 closed-ended and 1 open-ended) pertained to pet vaccination status and knowledge and use of rabies vaccination clinics in the county, reaction to a rabid animal, and choice of outlets to receive rabies-associated public health information. This is a subset of the questionnaire; results from other portions of the survey have already been published in a previous manuscript [11]. 

**Data analysis**—Data are presented in tables with frequencies and percentages. Chi-square testing using SAS [18] was used to compare frequencies for specific questions with an alpha value of ≤0.05. 

## 3. Results

**Socio-economic and demographic characteristics of respondents**—The overall ethnic/racial distribution of the 301 respondents was: 220 White non-Hispanic/Latino (Whites 75%); 33 Hispanic/Latino (11%); 40 African American (13%). Only 23 participants required a Spanish version of the survey. Hispanic/Latinos and African American respondents had lower income levels than Whites. Income varied by ethnic/racial groups, with 65% of the Hispanic/Latino respondents reporting earnings of less than $20,000/year while 77% of the White respondents reported earning more than $35,000/year. Hispanic/Latino and African American respondents were younger and had lived in the area for less time compared to Whites [11]. Hispanic/Latinos had lower education levels than African American and Whites, with 39% of the Hispanic/Latino respondents having only completed grammar school. College completion was eight times higher among White respondents (65%) than African Americans (8%).

**Pet ownership and care**—Overall, half of the respondents indicated owning one pet; however, only 30% of the Hispanic/Latino respondents owned a pet (Chi-square *p*-value <0.00001 for comparison between Whites and Hispanic/Latino and African Americans combined). Of the respondents that owned pets, 85% indicated that they vaccinated for rabies in the last 12 months. Reasons for no rabies vaccination were: vaccination is only done for outdoor pets, cost of the vaccine too high, pet was too young to vaccinate, or they could not catch the animal to have it vaccinated. African Americans (60%) and Hispanic/Latinos (89%) reported no knowledge of low-cost rabies vaccination clinics offered by Guilford County, whereas a larger percent of White respondents (65%) were aware of the clinics. More Whites (*n* = 93) than Hispanic/Latinos and African Americans combined (*n* = 9) were aware of the low-cost clinics run by Guildford County Animal Control (Chi-square *p*-value = 0.000472). Considering the people who were aware of the rabies clinics, 69% had heard about them from the local media (e.g., radio, television, or newspaper), but only 38% of them had vaccinated their pets at the low-cost clinics (Table 1).

When asked what they would do if they encountered a dog which they suspected had rabies, most respondents, regardless of ethnicity, indicated that they would call someone to handle the animal. Latinos indicated they would call Animal Control but did not have the number. Conversely, African Americans and Whites indicated they would call Animal Control, and did have the number (Table 2). A total of 30% of respondents indicated that they would call 911. The majority of respondents of all ethnic groups indicated that they would go to the emergency room if they were bitten by a dog. Similarly, the majority of the respondents indicated that they would report the offending dog to Animal Control (Table 2). 

A total of 53% of the White respondents indicated they would like to receive future information about rabies over the internet, whereas Latinos (43%) and African Americans (51%) preferred to receive the information by postal mail. When asked what would be the best way to deliver information about future rabies clinics, most respondents (35%) chose local media outlets such as local radio and TV (Table 3).

## 4. Discussion

Pet vaccination rates reported by respondents was surprisingly high (85%). In surveys when the public are asked about regulatory compliance without penalty, there is a tendency to respond what it is expected and not what actually is done. Therefore, the results may reflect a response bias—more specifically, an expectation bias. Pet owners know that they should be vaccinating their pets for rabies and report they have done it, even when they have not. The average one-year tags for vaccination in the state of North Carolina is 520 and for three-year tags is 445; these are tags issued by veterinary or animal control agencies [19]. The pet population in Greensboro is expected to be of the order of 53,000 dogs and 58,000 cats using the American Veterinary Medicine Association Pet Ownership Calculator (https://www.avma.org/KB/Resources/Statistics/Pages/US-pet-ownership-calculator.aspx). These numbers exceed the reported averages of rabies tags issues by veterinarians or animal control agencies.

Most Hispanic/Latino respondents indicated they were not aware of the rabies vaccination clinics offered by the county. Lack of information in Spanish is a possible explanation, in combination with a delivery system not favored by Hispanic/Latinos. The responses highlighted specific information outlets, giving public health officials a very clear direction on how to disseminate information. In NC there are a few Spanish-speaking radio stations, one television channel, and a couple of newspapers either sold at low cost or provided for free at stores selling food or products from Latin America and the Caribbean. Hispanic/Latinos frequent stores owned by other Hispanic/Latinos, and information regarding low cost/free rabies vaccination clinics and preventive information can be posted on information boards usually available at these places. 

Information regarding whom to contact if an animal suspected of having rabies is encountered should be made available to the public. Although most survey respondents indicated that they would report a rabid animal to Animal Control, they were unaware of the correct number to call. Many respondents (30%) indicated they would report their concerns to 911, especially after being asked if they knew the number for Animal Control. Animal Control could provide the public with educational resources such as multilingual information leaflets, refrigerator magnets, and calendar reminders to disseminate rabies information about reporting rabies suspicious animals, telephones to contact authorities, vaccination protocols and laws, and reporting an animal bite. Furthermore, 911 emergency respondents could be provided with a protocol for responding to calls about rabies while dispatching Animal Control. 

Our results suggest that outreach efforts would benefit from considering how ethnicity, income, and education impact the information outlets used by the public. Hispanic/Latinos and African Americans responded that they would like to receive information through postal mail, whereas Whites indicated that they would prefer the information be made available via the internet. These differences may reflect differential access to the internet, as only 51% of Hispanic/Latinos and 49% of African Americans have a home internet connection, compared to 66% of non-Hispanic/Latino Whites in 2011 in United States [20]. Similarly, 41% of those surveyed by Pew Research Center reported earning less than $30,000 and having internet access at home, and only 22% of people without high school diploma had home internet access [20]. The mean income for Hispanic/Latinos in our study was $19,000 a year, and had lower education levels than any other demographic group (39% did not have a high school diploma). Most African Americans in this study fared similarly to Hispanic/Latinos in income (less than $30,000/year); however, they reported a higher level of education (45% reported having a high school diploma and some college education). Half of the Hispanic/Latinos and African Americans reported access to a mobile internet connection. Many state and county websites are not designed to be accessed by phone, and present limited content when accessed by mobile devices. Websites enhanced for mobile access are a possible system to use adding to the other preferred methods to receive information [20]. Cellular phones could also be used to send information to the public about rabies, rabies vaccinations, and exposure notifications [21], particularly as these messages can be delivered to target segments of the population by creating geographical boundaries for message recipients.

Risk perception and aversion is associated with culture and complicated by socio-economic and demographic status. Language is one of the many factors affecting message decoding and interpretation, but is a very important one. Knowing the population at risk is fundamental to establishing a public health program for rabies and other zoonoses. Studies like the one presented offer a glimpse of where to start, not only in one county in NC, but other places where a public health campaign is needed. Our studies have prompted county public health officials throughout the country to request our survey instrument. Several officials have shared the need to better inform different segments of the population, and the lack of research efforts in the area of outreach and publicity of vaccination and other community-service oriented efforts.

The Centers for Disease Control and Prevention ‘Simply Put’ guide for creating educational materials was used as the base for the elaboration of educational materials on rabies and other zoonotic diseases [22]. The educational materials are available for public, Animal Control, and non-governmental and animal rescue organizations, as well as public institutions. A bilingual trifold was developed; this contains a cutout portion where the public can write the numbers of the local Animal Control and Public Health. The effectiveness evaluation of the educational materials is pending. Additionally, a bilingual rabies coloring book, various brochures, and leaflets were made available to the Guildford Animal Control after the study completion; radio and television appearances and short messages were published in Spanish-language local newspapers.

## Figures and Tables

**Table 1:** Frequency of answers regarding pet vaccination status and awareness and use or rabies vaccination clinics in Greensboro, NC, 2009.

Survey Questions and Possible Answers	Responses % (*n*)			
	Total Responses	Hispanic/Latinos	African Americans	Whites
Do you own or live with someone who owns a cat, dog, or ferret?				
Yes	58.19 (174)	28.13 (9)	50 (20)	64.38 (141)
No	41.81 (125)	71.88 (23)	50 (20)	35.62 (78)
Have these pets been vaccinated against rabies in the last 12 months?				
Yes, all of them	84.57 (148)	66.67 (6)	95 (19)	84.51 (120)
Yes, some of them	8.57 (15)	11.11 (1)	5 (1)	8.45 (12)
No	6.86 (12)	22.22 (2)	0	7.04 (10)
Why did you not vaccinate some of the pets?				
I only vaccinate pets that live outside	6.67 (1)	0	0	9.09 (1)
I only vaccinate pets who live inside	13.33 (2)	33.33 (1)	0	9.09 (1)
Is too expensive	13.33 (2)	33.33 (1)	0	9.09 (1)
The pet is too young	13.33 (2)	0	0	18.18 (2)
The pet is too old	6.67 (1)	0	0	9.09 (1)
Given three-year vaccination	20 (3)	0	100 (1)	18.18 (2)
I have no time to take the pets to the veterinarian	6.67 (1)	0	0	9.09 (1)
Cannot catch the animal	13.33 (2)	0	0	18.18 (2)
Lack of attention to pet’s vaccinations	6.67 (1)	33.33 (1)	0	0
Are you aware of the low-cost/free rabies clinics offered by Guilford County?				
Yes	60.34 (105)	11.11 (1)	40 (8)	65.96 (93)
No	39.66 (69)	88.89 (8)	60 (12)	34.04 (48)
Have you ever vaccinated your pets in the low-cost/free rabies clinics offered by Guilford County?				
Yes	38.1 (40)	50 (1)	71.43 (5)	34.41 (32)
No	61.9 (65)	50 (1)	28.57 (2)	65.59 (61)
How did you find out about the low-cost/free rabies vaccination clinics offered by Guilford County?				
Media (newspaper, radio, TV, internet, email)	68.75 (66)	100 (1)	42.86 (3)	70.59 (60)
Family or friends	23.16 (22)	0	28.57 (2)	22.62 (19)
Veterinarian	18.75 (18)	0	28.57 (2)	16.47 (14)
Shelter	5.21 (5)	0	0	5.88 (5)

**Table 2:** Frequency of answers regarding rabid animal reporting and first response after rabies exposure in Greensboro, North Carolina, 2009.

Survey Questions and Possible Answers	Responses % (*n*)			
	Total Responses	Hispanic/Latinos	African Americans	Whites
If you were to encounter a large dog you suspect has rabies in your neighborhood, what would you do?				
Try to capture the animal to try to help it	0.67 (2)	3.03 (1)	0	0.46 (1)
Try to scare the animal away	2.01 (6)	0	5.13 (2)	1.83 (4)
Try to kill the animal	2.34 (7)	0	2.56 (1)	2.74 (1)
I would do nothing	1 (3)	0	2.56 (1)	0.91 (1)
Call someone that can take care of it	93.65 (280)	96.97 (4)	89.74 (35)	93.61 (205)
If you have to call someone about a rabid animal, who would be the easiest for you to call?				
Family member, friend, or neighbor	0.68 (2)	0	0	0.92 (2)
Animal control (I have the number)	35.37 (104)	12.9 (4)	47.37 (18)	36.41 (79)
Animal control (I do not have the number)	31.97 (94)	61.29 (19)	31.58 (12)	27.65 (60)
Local Public Health Department (I have the number)	0.68 (2)	0	0	0.92 (2)
Local Public Health Department (I do not have the number)	0.68 (2)	0	2.63 (1)	0.46 (1)
Police/911	30.61 (90)	25.81 (8)	18.42 (7)	33.64 (73)
How would you respond to a dog biting your hand?				
Call a doctor	16.67 (50)	15.15 (5)	10 (4)	18.72 (41)
Care for the wound yourself	4 (12)	3.03 (1)	0	5.02 (11)
Go to the emergency room	60.33 (181)	69.67 (23)	77.5 (31)	55.25 (121)
Find the dog’s owner and ask for vaccination records	19 (57)	12.12 (4)	12.5 (5)	21 (46)
Would you report the dog to anyone?				
Yes	96.66 (289)	93.94 (31)	100 (40)	96.33 (210)
No	3.34 (10)	6.06 (2)	0	3.67 (8)
If you have to report the dog, who would you report to?				
Family member, friend, neighbor	1.71 (5)	6.45 (2)	0	1.4 (3)
Animal Control	71.92 (210)	51.61 (16)	84.21(32)	72.56 (156)
Local Public Health Department	5.82 (17)	9.68 (3)	5.26 (2)	5.58 (12)
Police/911	20.55 (60)	32.26 (10)	10.53 (4)	20.47 (44)

**Table 3:** Frequency of answers regarding rabies information outlet preference in Greensboro, North Carolina, 2009.

Survey Questions and Possible Answers	Responses % (*n*)			
	Total Responses	Hispanic/Latinos	African Americans	Whites
If you want to learn more about rabies, what would be the BEST way to deliver that information to you?				
Internet	47.98 (119)	32.14 (9)	32.43 (12)	53.11 (94)
Mail	34.27 (85)	42.86 (12)	51.35 (19)	29.94 (53)
Television	15.32 (38)	14.29 (4)	16.22 (6)	15.25 (27)
Radio	2.42 (6)	10.71 (3)	0	1.69 (3)
How would you like to be informed about future low cost/free rabies clinics offered by Guilford County?				
Media (newspaper, radio, TV, Internet, email)	35.34 (41)	75 (6)	20 (4)	35.63 (31)
Family of friends	17.24 (20)	0	20 (4)	17.24 (15)
Veterinarian	12.93 (15)	25 (1)	5 (1)	14.94 (13)
